# The clinical significance of cyclin B1 (CCNB1) in invasive breast cancer with emphasis on its contribution to lymphovascular invasion development

**DOI:** 10.1007/s10549-022-06801-2

**Published:** 2022-11-22

**Authors:** Abrar I. Aljohani, Michael S. Toss, Andrew R. Green, Emad A. Rakha

**Affiliations:** 1 Academic Unit for Translational Medical Sciences, School of Medicine, Nottingham Breast Cancer Research Centre, University of Nottingham Biodiscovery Institute, University Park, Nottingham, NG7 2RD UK; 2 Department of Clinical Laboratory Sciences, College of Applied Medical Sciences, Taif University, Taif 21944, Saudi Arabia; 3 Histopathology Department, Faculty of Medicine, Menoufia University, Shebeen El-Kom, Egypt; 4 Department of Histopathology, Nottingham University Hospital NHS Trust, City Hospital Campus, Hucknall Road, Nottingham, NG5 1PB UK

**Keywords:** *CCNB1*, Breast cancer, Progression, LVI, Prognosis

## Abstract

**Background:**

Lymphovascular invasion (LVI) is regulated through complex molecular mechanisms. Cyclin B1 (*CCNB1*) was previously determined as being associated with LVI using large cohorts of breast cancer (BC) and artificial neural network (ANN) technique. In this study, we aimed to assess the association between *CCNB1* and LVI, other clinicopathological and other LVI-related biomarkers at the molecular (RNA transcriptomic) and proteomic levels in BC.

**Methods:**

Two transcriptomic BC cohorts (*n* = 2834) were used to assess the association between the expression of *CCNB1* at the mRNA level and clinicopathological characteristics and patient outcome. Tissue microarrays (TMAs) from a well-characterised BC cohort (*n* = 2480) with long-term outcome were also used to assess the clinical significance of CCNB1 protein expression using immunohistochemistry.

**Results:**

High *CCNB1* mRNA expression was associated with aggressive tumour behaviour, including LVI, larger size, higher tumour grade, high lymph nodal stage, hormonal receptor negativity, HER2 positivity and poor clinical outcome (all *p* < 0.0001). Similarly, high CCNB1 protein expression was associated with higher tumour grade, hormonal receptor negativity and HER2 positivity (all *p* < 0.0001). Additionally, there was a significant association between *CCNB1*- and LVI-related biomarkers including N-cadherin, P-cadherin and TWIST2 at the transcriptomic and proteomic level. Multivariate analysis revealed that CCNB1 was an independent predictor of shorter BC-specific survival (HR = 1.3; 95% CI 1.2–1.5; *p* = 0.010).

**Conclusion:**

*CCNB1* is a key gene associated with LVI in BC and has prognostic value. More functional studies are warranted to unravel the mechanistic role of *CCNB1* in the development of LVI.

**Supplementary Information:**

The online version contains supplementary material available at 10.1007/s10549-022-06801-2.

## Introduction

The rate of breast cancer (BC)-associated mortalities has significantly increased over the past two decades [1], which is mainly related to metastasising disease to the other vital organs. Between 12 and 20% of early-stage BC patients will develop metastasis [2, 3]. The metastatic cascade consists of a complex stepwise manner and failure to complete any of these steps can stop the process [4]. Lymphovascular invasion (LVI), which refers to the presence of tumour emboli within the lymphatic and/or vascular spaces in the peritumoural invasive area, is considered as the initial and cornerstone step in the metastatic process. Despite the propensity of invasive BC cells to invade surrounding stroma, only those that can interact with endothelial cells and invade the vascular wall will develop LVI and complete metastatic spread [5, 6]. These LVI tumour emboli can migrate to distant organs, infiltrate and grow at the metastatic sites resulting in nodal or distance metastasis.

LVI is an important prognostic factor in cancers, including BC [7]. At the molecular level, upregulation of certain genes and downregulation of others can increase tumour invasiveness, migration and the ability to penetrate vascular walls and survive in the new environment [8–11]. As the molecular mechanisms underlying LVI are complex and overlap with many other related biological phenomena of carcinogenesis and progress, the primary steps of LVI can be explored via differential expression between LVI-negative and LVI-positive BC, mainly by virtue of pathways that promote LVI and the associated critical genes. The advances and development in bioinformatic techniques and high-throughput molecular methods allowed identification of key genes on a large scale, such as those linked with LVI [12].

Several studies have indicated that the presence of LVI in a primary tumour can determine appropriate treatment plans for BC [13–15]. Therefore, it is imperative to recognise the unique challenges presented by BC and find biomarkers to help with better management of cancer and optimise clinical outcomes for those patients.

Cyclins are proteins that activate certain cyclin-dependent kinases (CDKs) necessary for cell cycle progression [16]. Cyclin B1 (CCNB1) is a member of the cyclin family and a critical initiator and with quality control function in cellular division [17]. CCNB1 plays a key role in regulating and complexing with CDK1 to promote transition from the G2 to the mitotic phase of the cell cycle [18]. Increasing evidence indicates that CCNB1 is overexpressed in a variety of human malignancies, including colorectal, BC and prostate cancer [18–20]. Inhibition of *CCNB1* causes cell cycle arrest in various cell lines by altering the expression of G2/M cell cycle regulators [18]. *CCNB1* is also involved in the proliferation, migration, apoptosis, chemoresistance and metastasis of tumours [21–23]. We previously identified *CCNB1* as an overexpressed gene in BC with positive LVI using two large transcriptomic cohorts of BC, including the Molecular Taxonomy of Breast Cancer International Consortium (METABRIC) [24] and The Cancer Genome Atlas (TCGA) [25], using artificial neural network (ANN) methodology [26]. However, the exact role of *CCNB1* in the development of LVI, including protein expression levels and its association with LVI, the effect of other clinical and pathological confounders and the crosstalk with the other proliferation and LVI-related biomarkers, is still unknown. As such, it was necessary to gain insight into the role it plays in the development of LVI and BC outcomes in clinical settings by assessing its association with LVI at protein level. This study aimed to evaluate the association between CCNB1 at the transcriptomic and proteomic levels and LVI status, LVI-related biomarkers and other clinicopathological parameters using large well-annotated BC cohorts with long-term follow-up.

## Materials and methods

### Transcriptomic analysis study cohort

Using the METABRIC (*n* = 1980) and TCGA (*n* = 854) cohorts [24, 25], an assessment of the association between *CCNB1* mRNA expression and different variables, including tumour grade, tumour size, molecular subtypes, LVI-related biomarkers, and patient outcomes, was performed. The Illumina Human HT-12 v3 platforms (Illumina, Inc., San Diego, USA) were used to examine the extracted mRNA from primary tumour samples in the METABRIC. For TCGA data, the required information about clinicopathological parameters and RNASeqV2 data was obtained from cBioPortal [27, 28]. A subset (*n* = 288) of the METABRIC cohort was utilised to assess the correlation between mRNA and protein expression where data on the expression level of both parameters was available.

The METABRIC and the TCGA cohorts that were used in our previous study were used to evaluate the mRNA expression and its association with LVI status. Most patient demographics were similar in the TCGA and METABRIC cohorts. However, in the METABRIC cohort, LVI status has previously been evaluated for 1565 patients, including the Nottingham subset from the METABRIC cohort, using histological investigation of H&E-stained paraffin-embedded tissues. LVI status in Nottingham subset was determined by endothelial markers through IHC staining for CD31, CD34, and D2-40 [29]. However, the LVI was evaluated in the TCGA by assessing histological slides stained with H&E as no vascular IHC biomarkers were carried out for these samples. The clinical characteristics for the METABRIC and TCGA cohorts are shown in Supplementary Table 1. In regard to the patients’ cohort used in this study, LVI status was evaluated using both H&E-stained slides and IHC staining markers.

In addition, whilst in the METABRIC cohort, the overall distribution of intrinsic BC subtypes was assessed via prediction analysis of 50 genes using the PAM50 method, a technique based on RT-qPCR, the BC subtypes of the Nottingham cohort were identified using IHC profiling and the Elston–Ellis mitotic score [30]. Details of the clinicopathological factors of Nottingham cohorts are shown in Supplementary Table 2.

### CCNB1 protein expression

Sample tissues were obtained from well-characterised BC cohorts. This cohort contained 2480 primary invasive BC patients presented at the Nottingham City Hospital. Every patient’s clinicopathological profile was available, including age at diagnosis, size and nodal stage of the primary tumour, histological grade, LVI status and the Nottingham Prognostic Index (NPI). Oestrogen receptor (ER), progesterone receptor (PR) and human epidermal growth factor 2 (HER2) data were available for this cohort [31–34]. The BC molecular subtypes, luminal A (ER^+^ /HER2^−^; Ki67 < 10%), luminal B (ER^+^ /HER2^−^; Ki67⩾10%), HER2^−^ enriched (HER2^+^ regardless of ER status) and triple-negative BC (ER-, PR^−^ and HER2^−^), were characterised according to immunohistochemistry (IHC) profiles. Outcome data in terms of BC-specific survival (BCSS), in months, were available, defined as the time when the patient underwent surgery to when they died from BC. Patient treatment was based on the tumour features, NPI and the status of hormone receptors. Patients with ER^+^ tumour and high NPI scores (> 3.4) were given endocrine therapy. Those with the “good” NPI scores (≤ 3.4) were not given adjuvant therapy. Chemotherapy was given to premenopausal patients with moderate and poor NPI scores, whilst only hormonal therapy was given to postmenopausal patients with “moderate” or “poor” NPI scores. The classical treatments for patients without ER expression were cyclophosphamide, fluorouracil, and methotrexate. None of the patients in the study cohort received neoadjuvant therapy.

To gain more insight into the *CCNB1* molecular interactions, the correlation with epithelial–mesenchymal transition (EMT)-related markers, such as E-cadherin, N-cadherin, P-cadherin, TGFβ1, and TWIST2 [35, 36] was investigated. Supplementary Table 3 lists the cut-offs used to determine the expression levels of all these biomarkers.

### Tissue microarrays and CCNB1 antibody validation and immunohistochemical (IHC) staining

The primary mouse monoclonal anti-CCNB1 antibody (ab72, Abcam, UK)’s specificity was validated using western blot (WB) prior to staining with the IHC. MCF-7, SK-BR-3, and MB-MDA-231 (obtained from the American Type Culture Collection, Manassas, VA, USA) BC cell line lysates were used. In brief, 1:1000 and 1:15,000 primary antibody ratio and secondary antibody (IRDye 700CW Donkey anti-mouse) ratio were applied, respectively. The visualisation of the endogenous control marker was aided by the rabbit monoclonal anti-GAPDH primary antibody (1:5000) (ab181602, Abcam, UK) with IRDye 800CW Donkey anti-rabbit fluorescent secondary antibody (LI-COR Biosciences). The Odyssey Fc with Image Studio 4.0 (LI-COR Biosciences) was used to visualise the CCNB1 band, which showed a specific band at the expected molecular weight of 40 kDa (Supplementary Fig. 1).

**Fig. 1:**
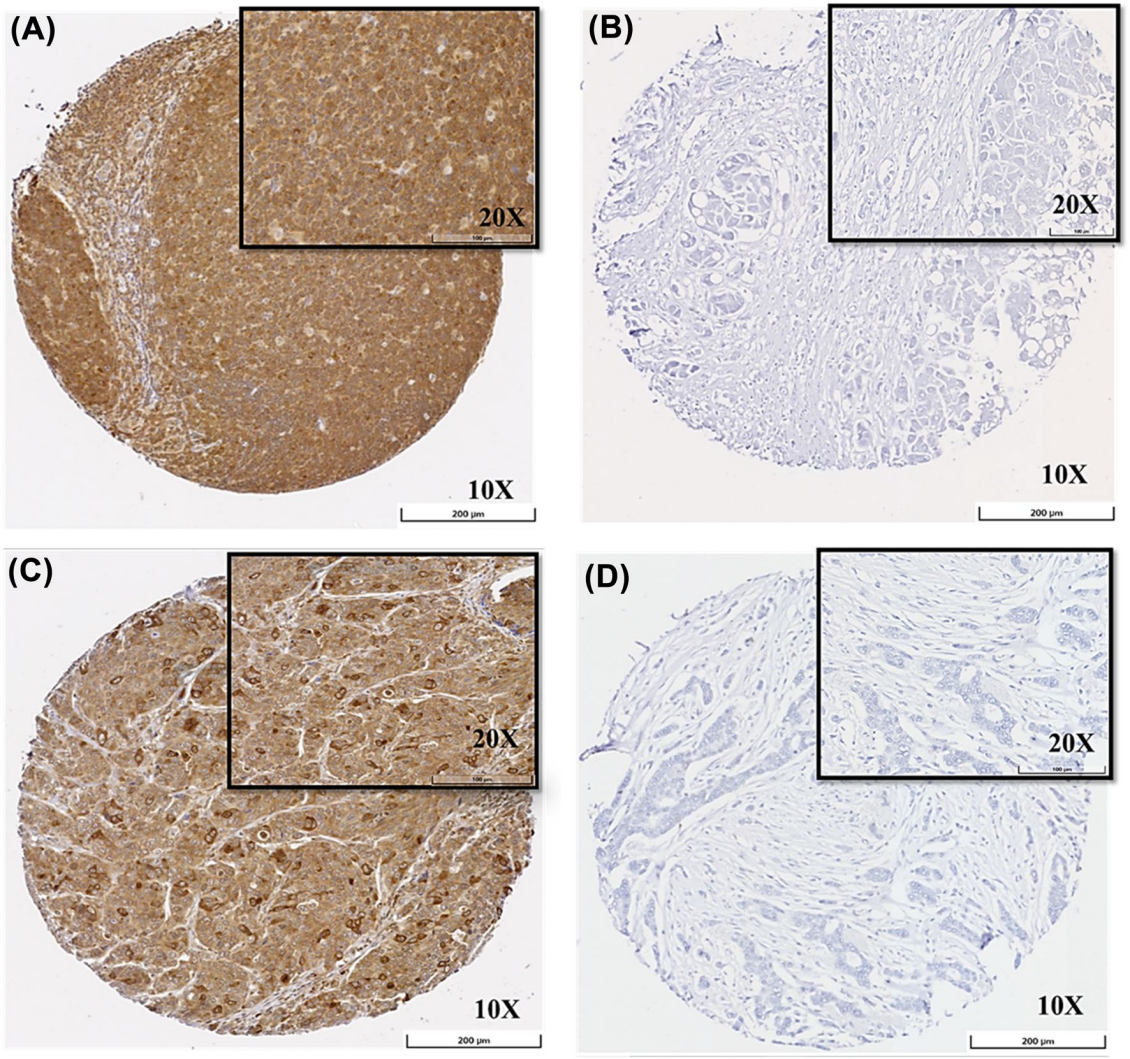
Cytoplasmic expression of CCNB1 protein in invasive breast cancer. **A** Positive control of tonsil tissue stained by CCNB1, **B** Negative control of liver stained by CCNB1, **C** Positive CCNB1 IHC expression and **D** Negative CCNB1 IHC expression. Magnification 10 ×. Scale bars = 200 μm. Inset, magnification 20 ×. Scale bars = 100 μm

The Grand Master® (3D HISTECH®, Budapest, Hungary) was used to prepare tissue microarrays (TMAs) from invasive BC tissues [35]. Using the Novocastra Novolink™ Polymer Detection Systems kit (Code: RE7280-K, Leica Biosystems, Newcastle, UK), the process of staining the TMAs by IHC was done on 4-μm TMA-thick sections. Antigen retrieval was performed (citrate buffer pH 6 at 1000 W for 20 min using microwave energy) following the manufacturers’ recommendations for this antibody. The dilution of the mouse monoclonal CCNB1 was done at 1:5000 ration in the Leica antibody diluent (RE AR9352, Leica Biosystems, UK), which was followed by a 15-min incubation at room temperature. Normal liver and tonsil tissues were used as negative and positive controls, respectively (Fig. 1A, B).

### CCNB1 protein expression assessment

TMA-stained sections were scanned into high-quality digital images, and this was carried out using a NanoZoomer scanner (NanoZoomer; Hamamatsu Photonics, Welwyn Garden City, UK) at × 20 magnification. The evaluation of CCNB1 cytoplasm expression was based on a semi-quantitative scoring by the modified histochemical score (H-score). During this evaluation, the staining intensity was multiplied by the positive cell percentage for every tissue. As such, a score ranging from 0 to 300 was produced [37]. The negative, weak, moderate and strong scores, which corresponded to a score index of 0–3, respectively, were used to assess intensity. The percentage of positive cells for every intensity went through a subjective assessment. The non-representative cores, such as cores in invasive tumour less than 15% of the core surface area and folded tissue during staining and processing, were not included in the scoring. IHC TMA slides were scored blindly and individually performed by a trained pathologist alongside the main researcher for at least 20% of the whole cohort under investigation. In the occurrence of a lower scoring concordance, the slides were re-scored and the differences between the scores discussed by the main researcher and a consultant pathologist. Excellent concordance of CCNB1 immunoscoring was observed between the two scorers (ICC = 0.9). CCNB1 protein expression data were not normally distributed and the cut-off for CCNB1 positivity was set using the median (100 H-score).

### Statistical analysis

IBM-SPSS statistical software 24.0 (SPSS, Chicago, IL, USA) was used for statistical analysis. *CCNB1* mRNA and CCNB1 protein expression continuous data were used to evaluate the correlation with clinicopathological parameters. The one-way analysis of variations (ANOVA) with post hoc Turkey multiple comparison tests was used to study the differences between three or more groups for parametric data. For non-parametric distribution, the ANOVA with Kruskal–Wallis test was used. In the METABRIC cohort, data about *CCNB1* mRNA expression were normally distributed and were categorised into low and high expression using cut-off generated from the mean. In the TCGA cohort, data about *CCNB1* mRNA expression were right skewed and were categorised using cut-off generated from the median. The difference between the two groups was evaluated using the Mann–Whitney test for non-parametric distribution and the Student *T* test for parametric data. The calculation of Spearman’s correlation coefficient was used to evaluate the association between continuous variables. The Kaplan–Meier curves were used to visualise the univariate analysis with the log-rank test. Cox’s proportional hazard regression model was developed to analyse multivariate survival. A *p value* below 0.05 was considered as statistically significant for all tests. The study was conducted following REMARK criteria [38] (Supplementary Table 4).

## Results

### *CCNB1* mRNA expression in BC

In METABRIC cohort, high *CCNB1* mRNA expression was observed in 922/1980 (47%), whilst in TCGA cohort it was 427/854 (50%). In both cohorts, a significant association was observed between high *CCNB1* mRNA expression and the presence of LVI (all; *p* < 0.0001) and other features characteristic of aggressive tumour behaviour, including large tumour size, high histological grade, hormonal receptor negativity (all; *p* < 0.0001) and HER2 positivity (*p* < 0.0001 in METABRIC and *p* = 0.006 in TCGA). In the METABRIC cohort, high expression of *CCNB1* mRNA was significantly associated with poor NPI and high LN stage (all; *p* < 0.0001).

Assessment of *CCNB1* mRNA in the intrinsic (PAM50) subtypes showed that high expression of *CCNB1* was correlated with luminal B, basal like, HER2 enriched, luminal A and normal-like subtypes in descending order (*p* < 0.0001) (Table 1). There was a weak correlation between *CCNB1* mRNA expression and CCNB1 protein expression (*r* = 0.136) when tested in the sub-cohort of METABRIC cases (*n* = 288).

**Table 1:** Statistical associations between *CCNB1* mRNA expression and clinicopathological parameters in the METABRIC (*n* = 1980) and TCGA (*n* = 854) breast carcinoma datasets

Parameters	*CCNB1* mRNA (METABRIC)			*CCNB1* mRNA (TCGA)		
	Number (%)	Mean rank	*p* value	Number (%)	Mean rank	*p* value
Patient age (year)			**0.041**			0.216
≤ 50	424 (21.4)	6.62		231(27)	444.66	
> 50	1556 (78.6)	6.55		623 (73)	421.14	
Tumour size			** < 0.0001**			** < 0.0001**
≤ 2 cm	622 (31.7)	6.47		239 (28)	362.98	
> 2 cm	1338 (68.3)	6.61		615 (72)	452.57	
Tumour grade			** < 0.0001**			** < 0.0001**
I	170 (9.0)	6.20		89 (11)	204.56	
II	770 (40.6)	6.43			327.36	
III	952 (50.3)	6.77		375 (46) 352 (43)	546.50	
Nottingham prognostic index (NPI)			** < 0.0001**	Not available		
Good	680 (34.3)	6.36				
Moderate	1101 (55.6)	6.67				
Poor	199 (10.1)	6.77				
Lymph node stage			**0.001**	Not available		
I	1035 (52.5)	6.51				
II	622 (31.5)	6.63				
III	316 (16.0)	6.66				
Lymphovascular invasion (LVI)			** < 0.0001**			** < 0.0001**
Negative	930 (59)	6.53		559 (65)	395.47	
Positive	635 (41)	6.66		295 (35)	488.20	
Oestrogen receptor (ER)			** < 0.0001**			** < 0.0001**
Negative	474 (23.9)	6.73		185 (22)	562.99	
Positive	1506 (76.1)	6.52		639 (78)	368.93	
Progesterone receptor (PR)			** < 0.0001**			** < 0.0001**
Negative	940 (47.4)	6.65		272 (33)	511.06	
Positive	1040 (52.6)	6.51		546 (67)	358.91	
Human epidermal growth factor receptor 2 (HER2)			** < 0.0001**			**0.006**
Negative	1733 (87.5)	6.54		567 (81)	340.36	
Positive	247 (12.5)	6.81		133 (19)	393.73	
PAM50 subtypes			** < 0.0001**	Not available		
Luminal A	718 (36.4)	6.28				
Luminal B	488 (24.7)	6.94				
HER2^+^ enriched	240 (12.1)	6.79				
Basal like	329 (16.7)	6.83				
Normal like	199 (10.1)	6.03				

*p* values in bold are statistically significant

### CCNB1 protein expression in BC

CCNB1 protein expression was observed mainly in the cytoplasm of invasive BC cells, with occasional cases showing minimal to weak nuclear expression, which were not sufficient to perform meaningful statistical analysis. The cytoplasmic expression levels varied from absent to strong (Fig. 1C, D). A high CCNB1 protein level (> 100 H-score) was observed in 1141/2480 (46%) of BC cases. A high CCNB1 protein level was significantly correlated with high tumour grade (including high pleomorphism scores, high mitotic count scores), poor NPI, hormonal receptor negativity (ER/PR) (all *p* < 0.0001) and HER2 positivity (*p* = 0.011). In the IHC subtypes, high expression of CCNB1 was associated with ER^−^/HER2^−^, HER2^+^, ER^+^/HER2^−^ high proliferation subtype, followed by ER^+^/HER2^−^ low proliferation subtype (*p* < 0.0001) (Table 2).

**Table 2:** Statistical associations between CCNB1 protein expression and the clinicopathological factors in the Nottingham BC cohort (*n* = 2480)

Nottingham BC cohort			
Parameters	CCNB1 protein		
	Number %	Mean rank	*p* value
Patient age (year)			0.093
≤ 50	843 (34)	1273.9	
> 50	1637 (66)	1223.3	
Tumour size			0.101
≤ 2 cm	1371 (55.3)	1261.6	
> 2 cm	1109 (44.7)	1214.5	
Tumour grade			** < 0.0001**
I	371 (20)	371	
II	875 (35.3)	875	
III	1234 (44.7)	1234	
Mitosis			
I	971(39)	1114.6	<0.0001
II	498 (20.1)	1210.4	
III	1011 (40.9)	1376.2	
Pleomorphism			** < 0.0001**
I	51 (2.1)	1112.3	
II	766 (30.9)	1038.9	
III	1663 (67)	1337.3	
Tubular formation			0.070
I	167 (8.6)	1256.3	
II	775 (31.3)	1191.7	
III	1538 (60.1)	1263.4	
Lymphovascular invasion (LVI)			**0.014**
Negative	1703 (69)	1264.1	
Positive	777 (31)	1188.8	
Lymph node stage			0.994
I	1535 (61.9)	1240.2	
II	706 (28.5)	1242.4	
III	239 (9.6)	1237	
Nottingham prognostic index (NPI)			** < 0.0001**
Good	769 (31)	1140.9	
Moderate	1299 (52.4)	1278.8	
Poor	412 (16.6)	1305.8	
Oestrogen receptor (ER)			** < 0.0001**
Negative	620 (25.1)	1404.5	
Positive	1848 (74.9)	1177.5	
Progesterone receptor (PR)			** < 0.0001**
Negative	1046 (43)	1281.2	
Positive	1382 (57)	1163.9	
Human epidermal growth factor receptor 2 (HER2)			**0.011**
Negative	2084 (86)	1197.9	
Positive	340 (14)	1301.8	
Triple negative			** < 0.0001**
No	1995 (82.4)	1164.3	
Yes	426 (17.6)	1429.9	
Immunohistochemistry subtypes			** < 0.0001**
ER^+^/HER2^−^ Low proliferation	1063 (46.4)	1078.8	
ER^+^/HER2^−^ High proliferation	785 (34.3)	1251.4	
Triple negative	426 (18.6)	1429.9	
HER2^+^	147(0.7)	1317.2	

*p* values in bold are statistically significant

### The association between CCNB1 expression and LVI-related biomarkers

To further evaluate the role of *CCNB1* in BC and their interactions with other biomarkers related to the various LVI-related cascades, the METABRIC and TCGA datasets were interrogated for the correlation between *CCNB1* and other genes involved in invasion, EMT and adhesion. Based on previous publications, *E-cadherin*, *P-cadherin*, *N-cadherin*, *TWIST2* and matrix metalloproteinases (*MMPs)* were selected [5, 35, 36, 39, 40].

Both transcriptomic cohorts (METABRIC and TCGA) showed a significant positive linear correlation between *CCNB1* mRNA expression and the expression of EMT-related genes, including *N-cadherin*, *GSK3B*, *TWIST1, TWIST2, ZEB1, ZEB2, NFKB1* and *CTNNB1*, whilst a negative linear correlation was observed with *E-cadherin*. In the TCGA cohort, a similar correlation was observed between *CCNB1* mRNA expression and the expression of *P-cadherin* and *TGFB1*. In addition, in both cohorts, *CCNB1* mRNA expression was positively correlated with the expression of MMP-related genes expression, including *MMP1, MMP7, MMP9, MMP12, MMP15* and *MMP20*. In the Nottingham cohort, high CCNB1 protein level showed a negative correlation with E-cadherin and a positive correlation with N-cadherin, P-cadherin and TWIST2 (Table 3).

**Table 3:** Correlations of CCNB1 expression with mRNA and protein expression of the epithelial–mesenchymal transition (EMT) and matrix metalloproteinase (MMP) related genes

Gene names	METABRIC cohort		TCGA cohort		Nottingham cohort	
	Correlation value	*p* value	Correlation value	*p* value	Correlation value	*p* value
EMT-related genes						
E-cadherin	− 0.090	** < 0.0001**	− 0.128	** < 0.0001**	− 0.091	**0.003**
N-cadherin	0.079	** < 0.0001**	0.096	**0.005**	0.083	**0.021**
P-cadherin	0.023	0.311	0.158	** < 0.0001**	0.145	** < 0.0001**
TGFβ1	0.014	0.522	0.300	** < 0.0001**	0.053	0.163
TWIST1	0.153	** < 0.0001**	0.157	** < 0.0001**	**Not available**	
TWIST2	0.289	** < 0.0001**	0.252	** < 0.0001**	0.079	**0.036**
ZEB1	0.203	** < 0.0001**	0.396	** < 0.0001**	**Not available**	
ZEB2	0.162	** < 0.0001**	0.259	** < 0.0001**		
NFKB1	0.264	** < 0.0001**	0.296	** < 0.0001**		
GSK3B	0.134	** < 0.0001**	0.122	** < 0.0001**		
CTNNB1	0.188	** < 0.0001**	0.178	** < 0.0001**		
MMP-related genes						
MMP1	0.300	** < 0.0001**	0.357	** < 0.0001**	**Not available**	
MMP7	0.068	**0.002**	0.113	**0.001**		
MMP9	0.116	** < 0.0001**	0.124	** < 0.0001**		
MMP11	0.035	0.115	0.036	0.290		
MMP12	0.195	** < 0.0001**	0.291	** < 0.0001**		
MMP15	0.103	** < 0.0001**	0.070	**0.042**		
MMP20	0.064	**0.004**	0.065	**0.057**		
MMP25	0.053	**0.018**	0.022	0.519		

### The association of CCNB1 expression and patient’s outcome

**Fig. 2:**
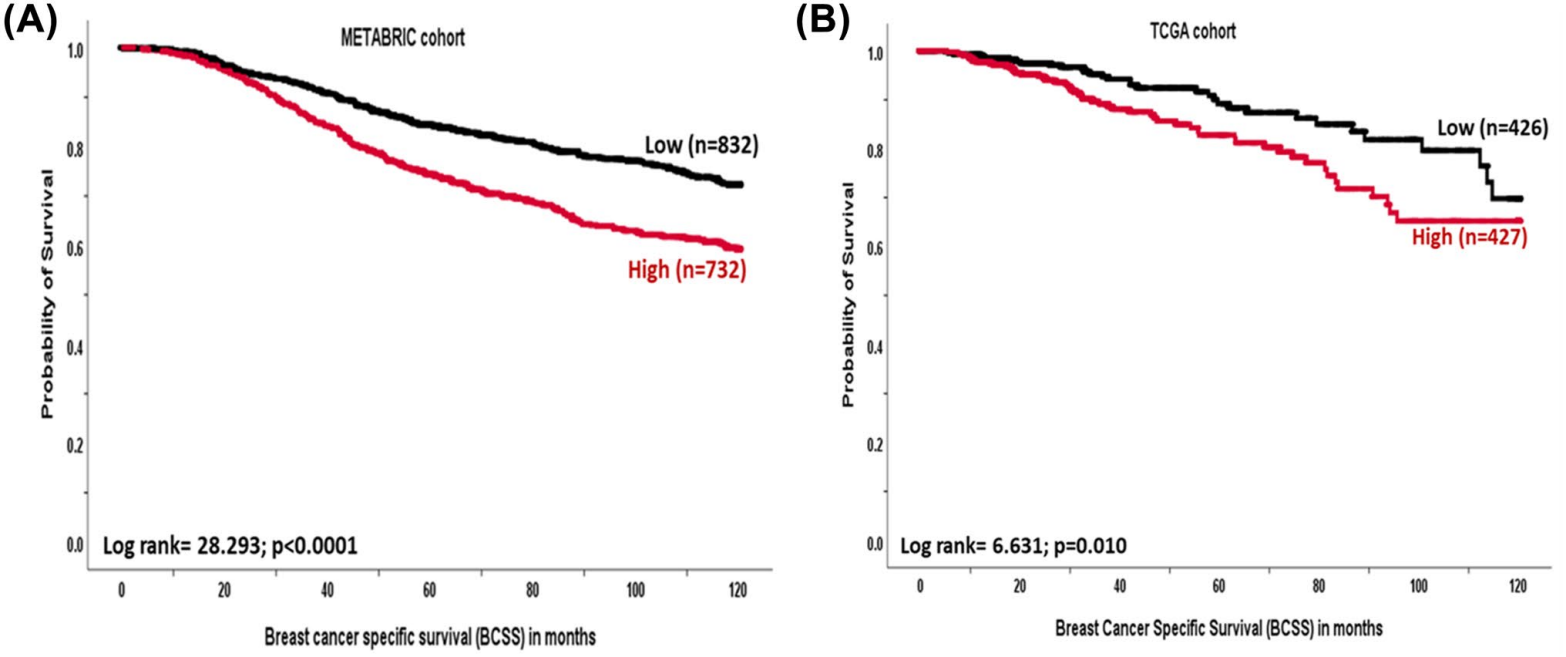
Kaplan–Meier survival plots showing the association between *CCNB1* mRNA expression and breast cancer-specific survival (BCSS) in (**A**) whole cohort (METABRIC) and **B** whole cohort (TCGA)

In the METABRIC cohort, survival analyses of *CCNB1* mRNA showed that *CCNB1* overexpression was significantly associated with shorter BCSS (*p* < 0.0001, Fig. 2A). Similarly, in the TCGA cohort, high *CCNB1* mRNA expression was associated with shorter outcome (*p* = 0.010, Fig. 2B). At the protein level, there was no significant association between cytoplasmic CCNB1 expression and patient outcome in the univariate analysis (Supplementary Fig. 2). However, when the overall expression was considered (cytoplasmic and nuclear), high protein expression was associated with shorter disease-specific survival (HR = 1.3, 95%CI 1.1–1.5, *p* = 0.002) which is consistent with the mRNA level.

In the METABRIC cohort, multivariate Cox regression analysis showed that *CCNB1* mRNA predicted poor BCSS survival independent of tumour size, LN stage, tumour grade and LVI (HR 1.5; 95% CI 1.2–1.8; *p* < 0.0001). In the Nottingham cohort, Cox regression analysis showed that high expression of CCNB1 was a significant predictor of shorter BCSS regardless of LVI status, tumour size, LN stage and tumour grade (HR = 1.3; 95% CI 1.1–1.5*;* *p* = 0.010) (Table 4).

**Table 4:** Multivariate Cox regression for predictors of breast cancer-specific survival (BCSS) and *CCNB1* mRNA expression in the METABRIC and TCGA cohorts and protein expression in the Nottingham BC cohort

METABRIC cohort				
Parameters	Hazard ratio (HR)	95% confidence interval (CI)		Significance *p* value
		Lower	Upper	
*CCNB1* mRNA expression	1.514	1.209	1.895	** < 0.0001**
Tumour size	1.448	1.104	1.899	**0.007**
Lymph nodal stage	1.788	1.537	2.080	** < 0.0001**
Tumour grade	1.304	1.066	1.595	**0.010**
Lymphovascular invasion (LVI)	1.393	1.100	1.764	**0.006**
TCGA cohort				
*CCNB1* mRNA expression	1.641	0.977	2.758	0.061
Tumour size	1.416	0.812	2.467	0.220
Lymph nodal	1.279	0.757	2.158	0.357
Tumour grade	1.079	0.740	1.573	0.692
Lymphovascular invasion (LVI)	1.836	1.118	3.014	**0.016**
Nottingham BC cohort				
CCNB1 protein expression (cytoplasmic)	1.300	1.101	1.500	**0.010**
Tumour size	1.439	1.217	1.701	** < 0.0001**
Lymph nodal stage	1.754	1.565	1.965	** < 0.0001**
Tumour grade	1.750	1.524	2.009	** < 0.0001**
Lymphovascular invasion (LVI)	1.423	1.200	1.688	** < 0.0001**
Nottingham BC cohort				
CCNB1 protein expression (cytoplasmic and nuclear)	1.181	1.008	1.385	**0.040**
Tumour size	1.4411	1.218	1.704	** < 0.0001**
Lymph nodal stage	1.752	1.564	1.963	** < 0.0001**
Tumour grade	1.693	1.478	1.938	** < 0.0001**
Lymphovascular invasion (LVI)	1.428	1.204	1.693	** < 0.0001**

*p* values in bold are statistically significant

### Discussion

One of the hallmarks of human cancer is abnormal cell cycle regulation [41]. Uncontrolled cell division is a necessary step in the progression of cancer. Several studies have found that cyclins, which orchestrate normal cell cycle, have abnormally increased expression in a variety of human malignancies [42, 43]. Cyclins are required to activate distinct CDKs at different stages of the cell cycle. Amongst the several cyclin/CDK complexes involved in cell cycle regulation, CCNB1/cdc2 is a well-studied complex that regulates G2/M phase checkpoint surveillance and is required for mitotic initiation [44, 45]. *CCNB1* is essential in checkpoint regulation, as its dysregulation is an early event in carcinogenesis [46]. *CCNB1* has been extensively studied in many solid tumours, such as lung [47], hepatic [48], and pancreatic cancers [20]. We previously identified *CCNB1* as a gene associated with LVI status using two large transcriptomic cohorts of BC and ANN methodology [26]. Briefly, the identification of the differentially expressed gene(s) between LVI positive and negative in the METABRIC [24] and the TCGA [25] BC cohorts was achieved using ANNs. To identify the enriched concordant biomarker set that is related to LVI, it was recommended to perform ANN-based neutral data mining on the genomic expression information obtained from the datasets identified early. Therefore, this followed the execution of the machine learning (ML) strategy grounded on the ANN and incorporated with concordance analysis executed in many Monte Carlo data splits [49]. This methodology efficiently eliminated over-fitting and false discovery whilst improving the identified biomarker generalisation. The concordant transcripts that have the least test error available in many loops for every group were identified by filtering the results. One of the top-ranked identified genes related to LVI positivity in both TCGA and METABRIC cohorts was *CCNB1* [26]. However, this is the first study, to the best of our knowledge investigating the association between *CCNB1*, LVI and LVI-related biomarkers in invasive BC.

From G0/G1 through the mid-S phase, *CCNB1* is relatively undetectable in cells; it becomes apparent in the cytoplasm in the late S phase. *CCNB1* levels rapidly increased in the perinuclear region of the cytoplasm as cells progressed through the G2 phase and it then appears in the nucleus during the mitotic phase [50, 51]. In this study, CCNB1 expression was observed in the cytoplasm in a large number of cases, whilst only a small number of cases showed nuclear staining. CCNB1 cytoplasmic localisation has been previously identified in some types of cancer, including BC [52]. During the late S/G2 phase of normal human cells, CCNB1/cdc2 complexes accumulate in the cytoplasm and must be translocated into the nucleus to initiate mitosis [53]. However, when DNA is damaged, CCNB1/cdc2 complexes are preserved in the cytoplasm, most likely to avoid premature mitosis [54]. On the other hand, cytoplasmic CCNB1 accumulation has been demonstrated to initiate mitosis by passing a p53-mediated G2/M checkpoint [55]. Cytoplasmic CCNB1 expression causes abnormal cell cycle progression at the G2/M checkpoint, enhancing genomic instability and malignant transformation [56]. This supports the potential role of the cytoplasmic expression of CCNB1 in BC.

Tumour metastasis is a multistep process that begins with the separation of cancer cells from the initial tumour mass and proceeds with intravasation, extravasation and the formation of new foci in a distant organ [57, 58]. The siRNA knockdown approach showed significantly reduced cell proliferation, colony formation and invasion when an endogenous CCNB1 was disrupted in oesophageal squamous cell carcinoma (ESCC) cells. Furthermore, the findings from animal models suggest that high expression of CCNB1 enhances invasive tumour growth in vivo and most likely leads to lung metastasis [21]. A study found that *CCNB1* overexpression provided cells with a greater capacity for transmigration through oesophageal carcinoma endothelium cells and human lung endothelium cells, which may have altered the cytoskeletal structure and promoted extravasation [21]. High expression of *CCNB1* resulted in decreased E-cadherin expression and increased N-cadherin expression, which induced EMT, an important mechanism in the metastatic cascade [21, 59]. High CCNB1 levels are associated with TWIST2 in ESCC, suggesting that TWIST2 might play a role in CCNB1-induced EMT [21]. This study showed that LVI-related biomarkers, such as E-cadherin, N-cadherin, P-cadherin and TWIST2, were significantly associated with CCNB1 at both mRNA and protein levels, which is consistent with the abovementioned findings. Furthermore, CCNB1 was correlated with MMPs biomarkers, such as MMP1, MMP7 and MMP9. The production of MMP extracellular matrix (ECM)-degrading enzymes increases cell escape from the main tumour tissue and subsequent invasion into tumour-adjacent tissues, such as epithelial cell strata and eventually lymphatic vessels [60]. Although the association between CCNB1 and other proliferation, migration and invasion biomarkers ranged from weak to moderate correlation, it was statistically significant which indicates that these markers are contributing to the same oncogenic pathway in the context of the LVI process. High expression of *CCNB1* results in the continuous cell cycle and division of cancer cells, promoting their migration and metastasis to distant sites [61, 62]. Uncontrolled cell division promoted by *CCNB1* could lead to gaining genetic instability and mutations that could affect other key genes for cellular migration and invasion that ultimately lead to LVI. Thus blocking this cascade from the early proliferative phase can stop these processes. These findings demonstrate that overexpression of CCNB1 may control one of the mechanisms driving LVI.

High expression of *CCNB1* mRNA in both the METABRIC and TCGA cohorts showed an association with large tumour size, high tumour grade, poor NPI, LN stage, LVI positivity, ER^−^, PR^−^ and HER2^+^. Similar findings were observed at the protein level; however, high expression of CCNB1 was not associated with presence of LVI at the protein level in contrast to the mRNA level. Such a disparity between mRNA and protein levels can be explained by various mechanisms. Because mRNA levels primarily determine protein levels, there will be variation between cellular mRNA and protein levels if the cell is undergoing long-term dynamic activities, such as continuous proliferation, which refers to the steady state of the cell [63]. *CCNB1* has been identified as a critical target gene for promoting tumour proliferation [64]. As a result of the proliferation induced by tumour cells, when *CCNB1* is highly expressed in a malignant cell, the cell may not be stable in the long term, leading to variation between mRNA and protein levels. Another factor that could contribute to the difference in the significance between LVI and CCNB1 at the mRNA and protein level is the methodologies that are used for quantifying and statistically analysing gene expression in the METABRIC and TCGA cohorts, as well as the ways for determining LVI in the transcriptomic and proteomic cohorts. For example, in the Nottingham cases, LVI status was determined using morphology and immunohistochemistry staining for D2-40 [29]. However, cBioPortal H&E-stained slides were utilised to determine LVI status in the TCGA cohort. Although H&E slides can be used to evaluate LVI, it might be difficult to distinguish LVI-negative cases [65]. The weak correlation between mRNA and protein levels could also explain the contradictory results in LVI between protein and mRNA.

Cross-talk between proliferation and its related markers and LVI and tumour invasion has been investigated. A published study [66] that investigated the mRNA and protein expression of other proliferation markers including Raf, MEK, p-MEK, ERK, and p-ERK in BC patients found that their levels were higher in the lymph node positive than in the node-negative group. The lowest levels of expression were noticed in normal breast tissue. The clinicopathological parameters, including tumour size, stage, and positive lymph node number, were found to be strongly associated with higher expression of Raf, MEK, p-MEK, ERK, and p-ERK. Additionally, these biomarkers were associated with poor outcomes [66]. Other studies which investigated proliferation-related markers in BC, including CCNB2 and Ki67, demonstrated that high expression of these biomarkers was associated with the features of aggressive tumour behaviour, such as LVI, large tumour size, and shorter survival [26, 67]. These studies provided further evidence to support the link between the expression of proliferation-related markers, such as CCNB1 and LVI and metastasis.

In transcriptomic cohorts, high expression of *CCNB1* was associated with worse outcomes which was also obvious at the protein level independent on other prognostic factors, including LVI status, tumour size, LN stage and tumour grade. Additionally, at the protein level, survival analysis with consideration of the overall CCNB1 protein expression (nuclear and cytoplasmic) revealed that high overall protein expression is associated with poor outcome which reflects the actual mRNA expression. This highlights the potential role of combined cytoplasmic and nuclear expression of CCNB1 in driving LVI and poor prognosis in BC. This was supported by the independent association of CCNB1 expression with poor BCSS in multivariate analysis. These findings were consistent with numerous reports that have shown inconsistent findings of the association between CCNB1 expression and prognosis in solid cancers [45, 47, 68]. Several studies using RT-PCR to analyse gene expression at the RNA level discovered that CCNB1 was highly associated with poor outcomes in solid tumours. However, the link between increased CCNB1 expression at the protein level and clinical prognosis in solid tumours remains unclear [45, 68]. These disparities indicate that more research is warranted to elucidate the underlying mechanism and function of CCNB1 in tumour development and prognosis in various tumour types.

Our findings suggest that CCNB1 is a potential therapeutic target for inhibiting LVI in BC and reducing the occurrence of metastatic disease. The results also suggest that CCNB1 might be a useful diagnostic tool to identify patients with positive LVI status in BC. The diagnostic utility of CCNB1 was previously described in other malignancies, including non-invasive bladder cancer and rhabdomyosarcoma [69, 70].

## Conclusion

Evidence from this study demonstrated that CCNB1 is important biomarker for invasive BC progression and has a potential role in LVI development. The exact functional and mechanistic effects of CCNB1 in LVI process require further investigations including in vitro and in vivo models to substantiate our findings.

## Supplementary Information

Below is the link to the electronic supplementary material.

Supplementary file1 (DOCX 22 kb)

Supplementary file2 (DOCX 414 kb)

## Data Availability

The authors confirm the data that have been used in this work are available on reasonable request.

## Declarations

### Research involving human and/or animal participants

This article does not contain any studies with human participants or animals performed by any of the authors.
