# Supplementary material for: The clinical significance of cyclin B1 (CCNB1) in invasive breast cancer with emphasis on its contribution to lymphovascular invasion development
Source: Breast Cancer Res Treat. 2022 Nov 22;198(3):423–35. doi: 10.1007/s10549-022-06801-2 (PMC10036284; doi:10.1007/s10549-022-06801-2)
Supplement: Supplementary file 2 — Supplementary file2 (DOCX 414 kb) [file 10549_2022_6801_MOESM2_ESM.docx]

**
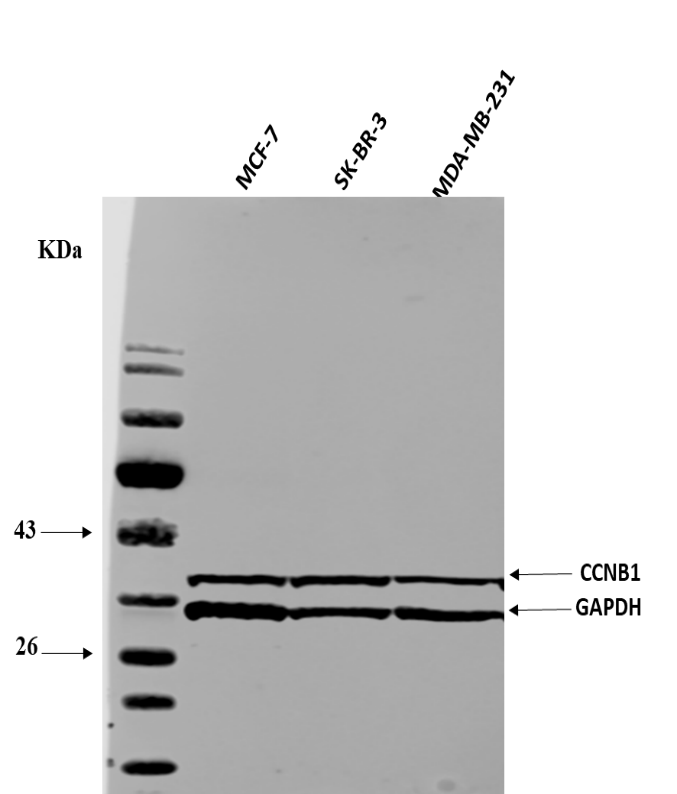
**

**Supplementary figure (1)** Western blotting reflecting the antibody specificity of CCNB1. GAPDH was used as a positive control.

**
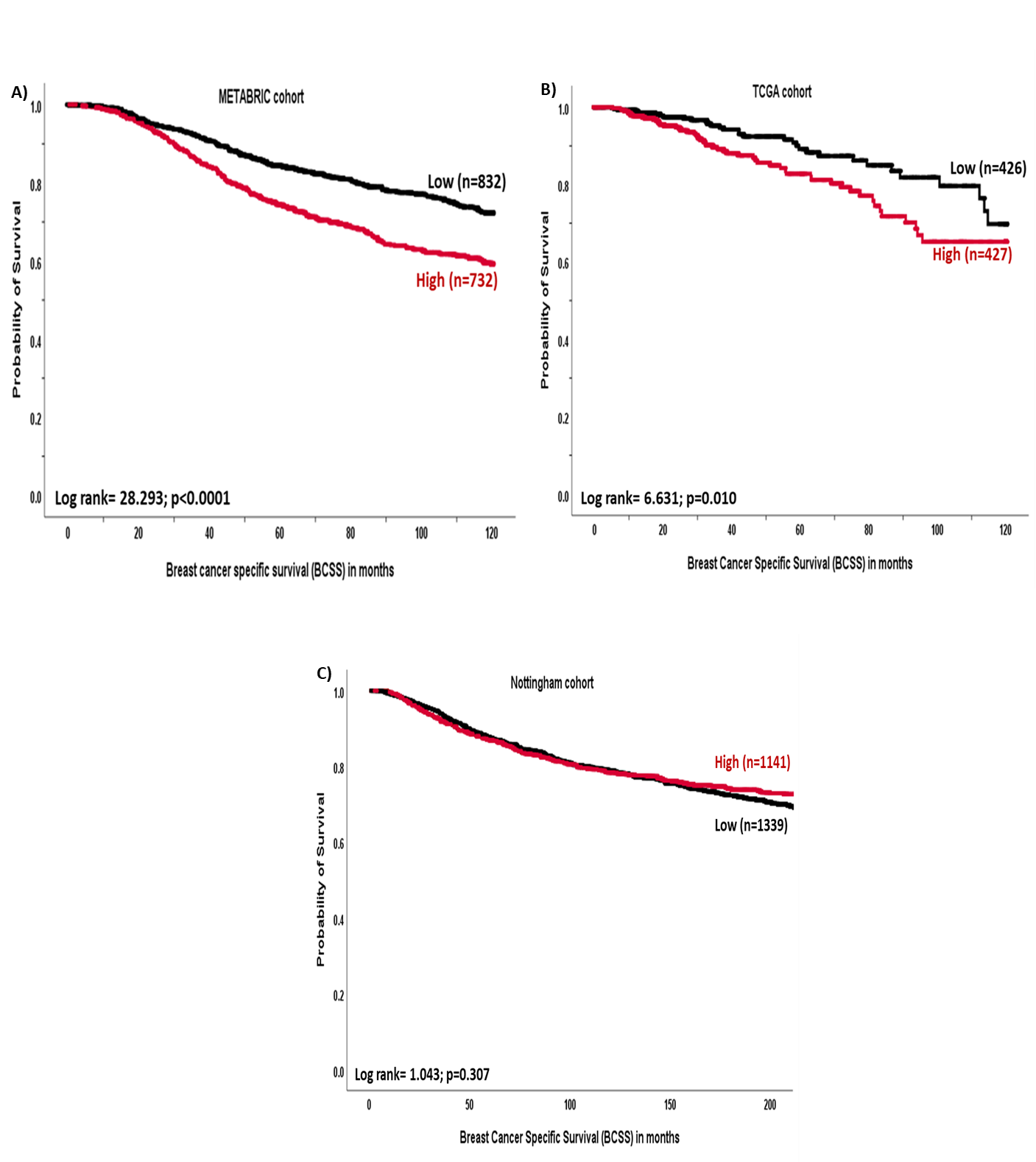
**

**Supplementary figure (2)** Kaplan–Meier survival plots showing the association in the Nottingham cohort between CCNB1 protein expression and BCSS.
